# 

**Published:** 1905-07-29

**Authors:** 


### NOTICE TO CORRESPONDENTS.

All MSS., Letters, Books for Review, and other matters intended for the Editor should be addressed The Editor, "The Hospital," The Hospital Buildings, 28 & 29 Southampton Street, Strand, W.C.

The Editor cannot undertake to return rejected MS., even when accompanied by stamped directed envelope.

**Notice to Advertisers and Subscribers.**

All Advertisements, Orders for Copies of The Hospital, and Business Communications should be addressed to **THE MANAGER (Not to the Editor),** The Hospital, 28 & 29 Southampton Street, Strand, London, W.C.

The Cost of Subscription, post free, is at follows:—Per week, 3½d.; per uarter, 3s. 3d.; per half-year, 6s. 6d.; per annum, 13s. Foreign and olonial:—Per annum, 19s. 6d., payable, in all cases, in advance.

**NOTICE.—Vol. XXXVII. of "The Hospital" October 1904 to March 1905), in handsome cloth binding, will shortly be on sale at the office of this Journal, price 10s. 6d. Cases for binding the Numbers contained in this Volume 2s. Index to Vol. XXXVII., 3½d., post free.**

**The Monthly Part for July is now ready. Price 1s., by post, 1s. 3d.**

### BOOKS RECEIVED.

Cassell and Co.

"The Care and Management of Delicate Children." By Percy Lewis, M.D.

J. B. Lippincott Company.

"International Clinics." Fifteenth Series. Vol. I.

### Medical Appointments.

**J. William Anderson**, M.B., Medical Officer to the Helston (Cornwall) Dispensary. **W. Blair Bell**, M.D., B.S.Lond., M.R.C.S.Eng., Assistant Gynæcological Surgeon to the Royal Infirmary, Liverpool. **Edgar G. Bulleid**, M.D., Clinical Assistant to the Chelsea Hospital for Women. **Carey Coombs**, M.D., B.S.Lond., Honorary Physician to out-patients, Royal Hospital for Sick Children and Women, Bristol. **Peter L. Daniel**, F.R.C.S.Eng., L.R.C.P.Lond., Assistant Surgeon to Charing Cross Hospital. **H. L. Eason**, M.D., M.S.Lond., Assistant Ophthalmic Surgeon to Guy's Hospital. **John Alfred Fox**, L.R.C.P.Lond., L.R.C.P.&S.Edin., L.F.P.S.Glasg., L.S.A. Lond., *pro tem.* Medical Officer for the Marazion District of the Penzance Union. **Octavius Hall**, L.R.C.P.&S.Edin., L.F.P.S. Glasg., D.P.H.R.C.P.S.Irel., Medical Officer of Health of Devonport. **Major F. P. Maynard**, I.M.S., to act as Ophthalmic Surgeon and Professor of Ophthalmic Surgery in the Medical College, Calcutta. **F. B. Mudd**, M.R.C.S., L.R.C.P.Lond., House Surgeon to the Johannesburg Hospital. **J. E. St. George Queely**, L.R.C.P.Irel., L.S.A., Medical Officer, Obenemasi G. M., Ashantee, West Africa. **V. H. Rutherford**, M.A., M.B.Cantab., Medical Officer to the Electrical Light and *X*-Ray Department, St. John's Hospital for Diseases of the Skin. **W. W. Saulter**, M.D., C.M., Clinical Assistant to the Chelsea Hospital for Women. **J. M. G. Swainson**, F.R.C.S., Resident Medical Officer at the North-Eastern Hospital for Children, Hackney Road, Assistant Surgeon to the Institution. **Frank Edward Tylecote**, M.D. Vict., Medical Registrar to the Manchester Royal Infirmary. **Robert Arthur Young**, M.D., F.R.C.P.Lond., Assistant Physician to the Hospital for Consumption and Diseases of the Chest, Brompton.

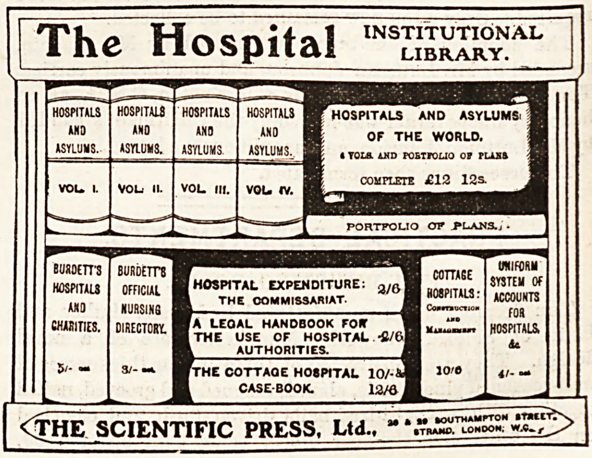

### BOOKS you SHOULD BUY, and WHY.

*Published by*
**THE SCIENTIFIC PRESS, LTD.**
28 & 29 Southampton Street,
Strand, London, W.C.

### HOW TO BECOME A NURSE.
**The Nursing Profession: How and Where to Train.**

Edited by Sir Henry Burdett, K.C.B.

2/- net, 2/4 post free.

Would-be Nurses will find this guide-book most helpful, as it contains a very complete List of Nurse Training Schools, with full details of Training, Premiums, Salaries, and Hours of Duty, &c., at each Institution, together with directions to whom application should be made in each case.

### ELEMENTARY ANATOMY AND SURGERY FOR NURSES.

By William McAdam Eccles, M.S.Lond., M.B., &c.

2/6 post free.

An elementary handbook designed to meet the exact requirements of Nurses from the standpoint of theoretical Anatomy and Surgery. The lectures are written clearly and concisely, and the work cannot fail to be of the greatest value to Nurses in the early stages of their training.

### ELEMENTARY PHYSIOLOGY FOR NURSES.

By C. F. Marshall, M.D., B.Sc., F.R.C.S.

2/- post free.

The idea of this book has been to deal with the subject in a concise manner, omitting abstruse problems of Physiology which are not essential to a Nurse actively engaged in hospital work.

### SURGICAL WARD WORK AND NURSING.

By Alexander Miles, M.D. Edin., &c.
3/6 net, 3/10 post free.

A very useful handbook for Nurses taking up Surgical Ward Work for the first time. It contains names and illustrations of the different instruments used in various operations, and explains very fully the duties of the Nurse with regard to operation cases.

### THE ART OF FEEDING THE INVALID.

NEW EDITION. With Special Chapter on Food for Consumptives. 1/6 post free.

This is an invaluable handbook in the Sick-room, the Household, and to all who have the care of Invalids and Convalescents. It contains chapters on Foods in Indigestion, in Corpulence, in Constipation and Diarrhœa, in Gout, in Liver and Kidney Diseases, in Consumption, in Convalescence and Chronic Illness, &c., with a large number of nourishing and appetising recipes.

### THE EXAMINATION OF THE URINE.

By J. K. Watson, M.D., Author of "A Handbook for Nurses." 1/- net, 1/1 post free.

This little work deals exclusively with the examination of the urine, and is written in the usual clear language that renders Dr. Watson's handbooks of such great help to Nurses.

**The Scientific Press, Ltd.**
THE NURSERY OF NURSING TEXT-BOOKS,
28 & 29 Southampton Street,
Strand, London, W.C.

Complete Catalogue

Post free on application.

### The NURSING MIRROR.

**The Nurse's Own Paper.**

**Price ONE PENNY Weekly.**

Of all Booksellers and Newsagents, or posted direct from the Office.

*Write for Subscription Rates to The Manager,*

**THE NURSING MIRROR, The Hospital Building, 28 & 29 Southampton Street, Strand, London, W.C.**

